# In‐use dissipation of technology‐critical elements from vehicles and renewable energy technologies in Vienna, Austria: A public health matter?

**DOI:** 10.1111/jiec.13571

**Published:** 2024-10-16

**Authors:** André Baumgart, Daniela Haluza, Thomas Prohaska, Simone Trimmel, Ulrike Pitha, Johanna Irrgeher, Dominik Wiedenhofer

**Affiliations:** ^1^ Institute of Social Ecology University of Natural Resources and Life Sciences Vienna Vienna Austria; ^2^ Department for Environmental Health, Center for Public Health Medical University of Vienna Vienna Austria; ^3^ Chair of General and Analytical Chemistry Montanuniversität Leoben Leoben Austria; ^4^ Institute of Soil Bioengineering and Landscape Construction University of Natural Resources and Life Sciences Vienna Vienna Austria

**Keywords:** emerging technologies, health hazards, industrial ecology, in‐use dissipation, material stocks and flows, rare earth elements

## Abstract

The rollout of electric vehicles and photovoltaic panels is essential to mitigate climate change. However, they depend on technology‐critical elements (TCEs), which can be harmful to human health and whose use is rapidly expanding, while recycling is lacking. While mining has received substantial attention, in‐use dissipation in urban areas has so far not been assessed, for example, corrosion and abrasion of vehicle components and weather‐related effects affecting thin‐film photovoltaic panels. Therefore, the question arises to which extent TCEs dissipate during use and which potential non‐occupational human health impacts could occur. We assessed the available information on urban in‐use dissipation and human health concerns and conducted exploratory modeling of in‐use technology stocks, in‐ and outflows, and in‐use dissipation of neodymium, dysprosium, lanthanum, praseodymium, cerium, gallium, germanium, and tellurium contained in 21 vehicle and renewable energy technologies, for Vienna, Austria. In prospective scenarios, TCE dynamics in a trend‐continuation vis à vis official city policy plans and a more ambitious transition scenario were then assessed. We find that electrifying the vehicle fleet without demand‐reduction is the main driver of TCE consumption, effectively doubling cumulative end‐of‐life outflows to 3,073 [2,452–3,966] t and cumulative in‐use dissipation to 9.3 [5.2–15.7] t by the year 2060. Sufficiency‐based measures could reduce demand and in‐use dissipation well below levels with continued trends, thus highlighting the need to combine decarbonization with demand‐reducing measures. These results help assess potential future in‐use dissipation dynamics and inform discussions about potential public health hazards associated with exposure to TCEs accumulating in the urban environment.

## 1 INTRODUCTION

Today, technology has become increasingly complex and depends on a range of technology‐critical elements (TCEs) (Graedel et al., 2015). In 2024, the European Commission enacted the Critical Raw Materials Act to ensure a secure and sustainable supply of such critical raw materials (European Commission, 2023b). TCEs are extensively used in electric vehicles, renewable technologies, and various electronics (Cobelo‐García et al., 2015; Zhang et al., 2023). These elements are regarded as critical both due to their fundamental importance to new technologies and due to supply insecurities arising from geopolitical shifts and short‐ to medium‐term potential shortages from mining (Filella, 2020b; Goodenough et al., 2018). TCEs encompass lanthanides, commonly known as rare earth elements (REEs), as well as platinum‐group elements (PGEs) and some assorted elements. Their utilization ranges from lithium (Li) in Li‐ion batteries (Junne et al., 2020), neodymium (Nd) in magnets, antimony (Sb) used as a catalyst for polyethylene terephthalate (PET) production (Filella, 2020a), to bismuth (Bi) in alloys, pigments, or cosmetics.

As TCEs are ever more prevalent in industrial and residential technologies, investigating the mechanisms, pathways, and long‐term consequences of human TCE exposure within urban contexts becomes necessary (Cobelo‐García et al., 2015). Despite substantial concerns expressed by the medical community (Cobelo‐García et al., 2015), research on human health risks due to accumulation of TCEs in the environment beyond lab studies is still scarce (Guhl, 2023; Gwenzi et al., 2018). Most studies focused on occupational exposure, for example, in mining, manufacturing, or recycling sites, where workers and residents can be substantially exposed to TCE pollution (Henríquez‐Hernández et al., 2018; Pagano et al., 2019; Takyi et al., 2021). Less literature deals with environmental TCE concentrations in sites where anthropogenically increased levels cannot be attributed to the primary extraction or processing of TCEs. Although TCEs occur in the earth's crust naturally, the highest TCE concentrations can be found around mining sites (Li et al., 2013; Traore et al., 2023). However, anthropogenically increased TCE levels have also been detected in urban areas (Liu et al., 2023; Yuan et al., 2018).

Therefore, interdisciplinary assessments of potential emission sources, environmental contamination, human exposure, and toxicity thresholds are necessary, to understand if there is an emerging public health issue requiring pro‐active action (Cobelo‐García et al., 2015). In the present study, we address aspects of this challenge by scoping how TCE use and potential in‐use dissipation can be modeled for those TCEs where a sufficiently solid understanding of the causality of in‐use dissipation is available. Addressing this research gap is crucial for developing effective strategies and regulatory measures to mitigate the potential health and environmental risks associated with TCEs in urban spheres. With themes such as critical resource consumption, reduction of unrecoverable material losses, sustainable transition technologies, and human health impacts, this issue is therefore relevant to several Sustainable Development Goals, in particular, Targets 3.9 “Reduce illnesses and deaths from hazardous chemicals and pollution,” 9.4 “Upgrade all industries and infrastructures for sustainability,” and 11.6 “Reduce the environmental impacts of cities” (United Nations General Assembly, 2015).

Previous work focused on TCE extraction, supply chains, and recyclability potentials (Du & Graedel, 2011; Filippas et al., 2021; Fishman et al., 2018; Nuss & Blengini, 2018; Peiró et al., 2013; Xiao et al., 2022; Yao et al., 2021). While knowledge on TCE accumulation and human exposure at extraction sites and landfills is increasing, material losses during the use phase of technological products and components, which often occur “by design” (Ciacci et al., 2015) have received comparatively less attention (Ciacci et al., 2016, 2015; Helbig et al., 2020; Mleczek et al., 2021; Zimmermann & Gößling‐Reisemann, 2013).

Different terms have been used to refer to such losses (Müller et al., 2014). We herein refer to them as “in‐use dissipation,” as used in Ciacci et al. (2015). Further, in‐use dissipation is understood as dissipative flows to the environment occurring during the use phase of products. This complements other types of material losses, such as those occurring mainly within the technosphere, that is, non‐functional outputs from the value chain or non‐functional recycling (Beylot et al., 2020), as more commonly investigated in life cycle assessments.

A significant share of TCEs is used in the mobility and energy sectors (Moss et al., 2013; Ortego et al., 2020). For example, more than 70% of Nd is currently used in transportation and wind power (Ciacci et al., 2019), mainly in NdFeB magnets, which also include other REEs. The demand for such technologies is rapidly increasing, partially also due to political directives aimed at mitigating climate change (European Commission, 2019, 2023a). For example, in 2022, more than 22% of newly registered cars in Austria are battery and plug‐in electric vehicles, slightly higher than the EU average of just below 22% (EEA, 2023). The share of other elements such as gallium (Ga), germanium (Ge), and tellurium (Te) in transportation and renewable energy technologies is lower according to Ciacci et al. (2015). However, these elements are nevertheless relevant from a public health perspective, as the causality of the in‐use dissipation from these technologies is much more comprehensible as opposed to their use in, for example, computer technologies. With increased utilization and in‐use stock accumulation comes potentially increased dissipation of TCEs to the environment (Filella & Rodríguez‐Murillo, 2017). Dissipation occurs across all life cycle phases, from extraction, manufacturing, and use to waste management including recycling or deposition, where TCEs may migrate to the air, soils, or waterways (Cobelo‐García et al., 2015). Above certain levels, individual TCEs like Nd, Ga, Ge, Te, and lanthanum (La) can negatively impact human health (Brouziotis et al., 2022; Sun et al., 2017; White & Shine, 2016) and ecosystem functioning (Balaram, 2019; Cobelo‐García et al., 2015).

In‐use dissipation can occur due to various reasons. In the case of vehicles, TCEs such as Nd and praseodymium (Pr) can dissipate to the environment via abrasion and corrosion (Ciacci et al., 2015; Mleczek et al., 2021). In the case of photovoltaics (PVs), TCEs such as Ga can be washed out by rainwater after panels become damaged by hail, or because they are incorrectly assembled (Celik et al., 2018; Nain & Kumar, 2020; Zapf‐Gottwick et al., 2015).

Evidence on possible toxicological hazards due to TCE accumulation in urban environments where non‐occupational exposure may occur is rare (Cao et al., 2016; Henríquez‐Hernández et al., 2020; Qvarforth et al., 2022; Yuan et al., 2018). A substantial body of literature showed possible public health and environmental implications of TCE mining and processing, as well as end‐of‐life (EoL) phases (Li et al., 2013; Nain & Kumar, 2020; Pagano et al., 2019; Yin et al., 2021). Consequently, the state of research on current and future in‐use dissipation of TCEs in urban environments and the related toxicological implications are scarce from a public health as well as a sustainability perspective.

Therefore, this study aims to model TCE accumulation, outflows, and in‐use dissipation under various decarbonization pathways, for the urban system of Vienna, Austria. The following research questions are addressed: *What is the current state of knowledge regarding TCE in‐use dissipation? What is the current size of TCE stocks, inflows, outflows*, *and in‐use dissipation? Which increase in quantities can be expected due to the mobility and energy transition in Vienna? To what extent might TCEs accumulate in the urban environment due to in‐use dissipation, and what is their toxicological relevance in humans?*

For this purpose, we modeled how official city plans as well as additional demand‐reducing strategies could affect TCE inflows, outflows, and in‐use dissipation and discuss possible toxicological implications of environmental accumulation. The city of Vienna was chosen as a case study site due to the Smart Climate City Strategy Vienna being recently passed (Stadt Wien, 2019), which details various targets such as fleet electrification or the expansion of installed capacity of renewable energy carriers. With almost 4,800 capita per km^2^ in 2023 (Stadt Wien, 2023) and 11,075 passenger kilometers traveled in 2015 (Gassner et al., 2020), Vienna is a densely populated, high‐traffic area where a higher degree of contact between the resident population and TCE pollution can be expected.

## 2 METHODOLOGY AND DATA

This study applies a bottom‐up, stock‐driven modeling approach (Saidani et al., 2019; Wiedenhofer et al., 2019) to quantify TCE stocks, inflows as gross additions to stock, and outflows separated into EoL and in‐use dissipation. Prospective exploratory scenarios were developed from 2020 to 2060 because official city strategies fall within this timeframe and because assessing technological change beyond this period is out of scope herein. A “trends” scenario continues current trends. Specific inputs such as future vehicle fleet composition or modal splits are modified based on decarbonization and circular economy policies to create transformation scenarios with varying intensity.

### 2.1 Modeling scope and system definition

**FIGURE 1:**
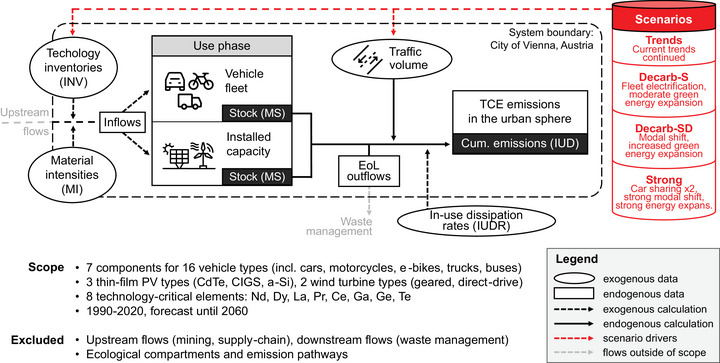
System definition and scope. Trends, scenario with continued trends; Decarb‐S, decarbonization with official targets with supply‐side measures; Decarb‐SD, decarbonization with official targets with combined supply‐ and demand‐side measures; Strong, scenario with measures going beyond official targets (see Section 2.3 for further information on scenarios). EoL, end‐of‐life; MS, material stock; PV, photovoltaic; TCE, technology‐critical element. Nd, neodymium; Dy, dysprosium; La, lanthanum; Pr, praseodymium; Ce, cerium; Ga, gallium; Ge, germanium; Te, tellurium.

The administrative boundary of Vienna, Austria, serves as a system boundary (Figure 1). No upstream (mining, production of technologies), or downstream (recycling facilities, deposition) activities outside of the city boundary are modeled. The scope of the modeling encompasses the eight TCEs Nd, dysprosium (Dy), La, Pr, Ce, Ga, Ge, and Te. On the one hand, these were chosen due to their criticality. The selected elements are commonly found in emerging technologies highly relevant for a sustainable transition but are subject to potential supply shortages (Ciacci et al., 2015). Second, scientific understanding regarding their anthropogenic distribution and associated health impacts remains vague compared to other TCEs. For example, while lithium, cobalt, nickel, and PGEs are considered critical as well; these materials have been investigated to a significantly higher degree (e.g., Hao et al., 2017; Henckens & Worrell, 2020; Huang et al., 2014; Su et al., 2023; Ziemann et al., 2012). While there is a wide spectrum of different applications of these TCEs, the focus of this study is on vehicles, PV, and wind power, due to their high relevance for decarbonization policies and as the causal dissipation mechanisms could be identified from the literature. The model covers 21 technology types in the mobility and energy sectors. Vehicle technologies include internal‐combustion engine (ICEV), hybrid‐electric (HEV) and battery‐electric (BEV) cars, ICEV and BEV trucks, motorcycles and buses, as well as e‐bikes and e‐scooters.

Various components of vehicles are distinguished, including motors, batteries, autocatalytic converters, brake pads, tire treads, front and rear axles, wheels, electronic systems, and various electronic accessories. For the energy sector, cadmium‐telluride (CdTe), copper‐indium‐gallium‐selenide (CIGS), and amorphous silicon (a‐Si) thin film PV, as well as geared and direct drive wind power engines were differentiated. Other, more commonly used PV technologies were not included as they do not contain any of the studied TCEs. Further hypothetical future technological changes were not considered in this study.

TCE stocks and flows from 1990 to 2020 were retrospectively modeled based on official data on vehicle fleets, traffic volumes, and installed capacity. Future stocks were prospectively modeled based on population‐coupled future traffic volume estimates. Exogenous model input data for technology inventories (fleet size or installed capacity), as well as TCE contents and in‐use dissipation rates, were sourced from literature to model TCE stocks, inflows, and in‐use dissipation (for details, see Section 2.4).

### 2.2 Modeling TCE stocks and flows

Following a stock‐driven modeling approach, TCE stocks and flows were modeled using material intensity (MI) factors in combination with statistical data (see Section 2.4 for data sources). Static MI factors were used, as no prospective changes in technologies or material efficiency were considered in this study.

For the calculation of stocks and flows, the programming language R was used. To quantify stocks, Equation (1) was used, where *MS* is the total TCE stock in a given year, *MI* is the material intensity, *INV* is the technology inventory or functional unit, *e* is the investigated TCE, *c* is the technology or technology component, and *t* is the corresponding year. For vehicles, the functional unit is a unit of component or vehicle (kg/unit). For PV and wind power, the installed capacity (MW) serves as the functional unit instead:

(1) $$MS_{e,t}=\sum_{c}\left[MI_{e,c}\times \;\mathrm{IN}V_{c,\;t}\right]$$

In‐use dissipation *IUD* (Equation (2)), that is, the share of total TCE mass lost in a year, was calculated independently of stocks as the flow of material is associated with other factors such as traffic volume and installed capacity. *vkm* is the vehicle kilometers (vkm) traveled within a year*, LTvkm* is the vkm driven during the lifetime, *LT* is the lifetime of a technology or technology component, and *IC* is the annual installed capacity. *IUDR* refers to the in‐use dissipation rate, that is, the material dissipated during use during the lifetime of a technology. Thus, the total TCE mass dissipated during the lifetime of use was first divided by the total distance traveled (vehicles) or technology‐specific product capacity (PV and wind power), thereby producing a dissipation factor per function unit. Multiplied with total traffic or installed capacity in a given year, we then computed annual in‐use dissipation. As shown in Equation (2), the total *IUD* is then calculated as the sum of individual vehicle and renewable energy *IUD* (first and second part of the equation):

(2) $$\mathrm{IU}D_{e,t}=\sum_{c}\left[\frac{MI_{e,c}\times \mathrm{IUD}R_{e,c}}{\mathrm{LTvk}m_{c}}\;\times \;\mathrm{vk}m_{c,\;t}\right]+\sum_{c}\left[\frac{MI_{e,c}\times \mathrm{IUD}R_{e,c}}{LT_{c}}\;\times \;IC_{c,\;t}\right]$$

As we are interested in actual distance traveled, passenger kilometers (pkm) and tonne kilometers (tkm) taken from Gassner et al. (2020) first had to be converted to vkm using vehicle‐specific occupancy rates (see Table SI‐2). For PV, in‐use dissipation rates were only used for a specific share of panels that were assumed to break and thereby allow for TCE dissipation during the use. Since age composition data are not available for the vehicle fleet and installed capacity in Vienna, we opted for a leaching lifetime model approach (van der Voet et al., 2002; Wiedenhofer et al., 2015) to approximate annual EoL emissions by dividing the material stock (MS) in a given year by the assumed lifetime (LT). Secondary equations for inflows and EoL outflows of TCEs, as well as a summary table of all variables with respective units, can be found in supplementary information S1.

### 2.3 Definitions for the exploratory prospective scenarios

Table 1 provides an overview of measures assumed for the trend continuation (“trends”) and three prospective scenarios. Two scenarios (Decarb‐S and Decarb‐SD) are based on official targets defined in the Smart City strategy (Stadt Wien, 2019) and are differentiated to better distinguish the effects between supply‐side and demand‐side measures. Vehicle fleet and installed capacity between the baseline and target year were calculated using linear interpolation. Both Decarb‐S and Decarb‐SD cover supply‐side measures such as the electrification of newly registered personal vehicles and an expansion of the installed capacity of PV to 800 MW by 2040. However, Decarb‐SD integrated supply‐ with demand‐side measures by introducing a modal split shift toward 85% public and active mobility. The “strong” scenario encompasses more ambitious combined decarbonization measures such as an increased modal split shift (95%), a doubling of PV expansion by 2060 compared to Decarb‐S, and a doubling of car sharing by 2050. A more detailed description of scenario differences can be found in supporting information S1.

**TABLE 1:** Definition of scenarios and scenario assumptions.

Scenario assumptions	Trends	Decarb‐S	Decarb‐SD	Strong
	Continued trends	Decarbonization with supply‐side measures	Decarbonization with combined supply‐ and demand‐side measures	Far‐reaching scenario with stronger measures
Trends continued	•			
Electrification of personal vehicles by 2050^1^		•	•	•
Photovoltaic capacity expansion to 800 MW by 2040^1^		•	•	•
Modal shift to public active by 2050 (85%)^1^			85%	
Modal shift to public active by 2050 (95%)				95%
Doubling of car sharing doubled by 2050				•
Electrification of all new vehicles by 2060				•
Doubling of PV expansion by 2060				•
Wind power expansion until	2030	2040	2050	2060

^1^ Assumption based on official targets currently envisioned in the Smart City strategy (Stadt Wien, 2019).

To quantify TCE stocks and flows from 1990 to 2020, Equation (1) was applied, using statistical inventory data for each year *t*. To estimate future stocks and flows in vehicles, first, future traffic volume was calculated by coupling historic pkm and tkm with population forecasts. Future fleet additions were then derived by coupling historic fleet additions with estimated future traffic volume until 2060. By applying a leaching lifetime model, the future vehicle fleet was then cumulatively calculated by adding and subtracting annual fleet additions and removals, respectively. Analogously, the future installed capacity of PV and wind power was calculated based on annual capacity additions and removals.

### 2.4 Data sources

In total, 45 literature sources were used to assemble MI factors for technological components of mobility and energy technologies (see supporting information S2 for the complete dataset with sources). For all vehicles except for buses, e‐bikes, and e‐scooters, MI factors were compiled for each component; consequently, these vehicles’ total TCE content is the sum of the TCE contents of their components. Component‐specific MI factors for motors, front axles, wheels and brakes, brake pads, tire treads, selector mechanisms, electronic systems, accessories, and vehicle bodies were differentiated. Furthermore, for ICEV and HEV, autocatalytic converters, and HEV, nickel‐metal hydride batteries were distinguished. For other vehicles, MI factors were available per unit only. MI factors for energy systems (PV and wind power engines) were available per MW of installed capacity.

Lifetime assumptions *LT* for the leaching model, in‐use dissipation rates *IUD*, breakage rates, and other model assumptions can be found in Table SI‐1. Due to a lack of data on the in‐use dissipation of TCEs in other vehicles, PV panels, and wind power engines, a rate of 2% similar to that of cars was assumed. Because only two sources could be identified for deriving in‐use dissipation rates, in‐use dissipation modeled herein should therefore be seen as a first rough estimate highlighting its possible scale, rather than a precise estimate.

### 2.5 Uncertainty

To account for uncertainties regarding model inputs, ranges for key parameters were created. For MI factors, the interquartile range, that is, the 25th and 75th percentiles, were used as lower and upper bound estimates. In addition, in‐use dissipation factors, for which the body of literature is unfortunately very small, as well as vkm per lifetime were varied by ± 10% for lower and upper bound estimates, respectively. To quantify maximum ranges, either all lower or all upper bound input values were used.

## 3 RESULTS

### 3.1 TCE stocks and flows in the year 2020

**FIGURE 2:**
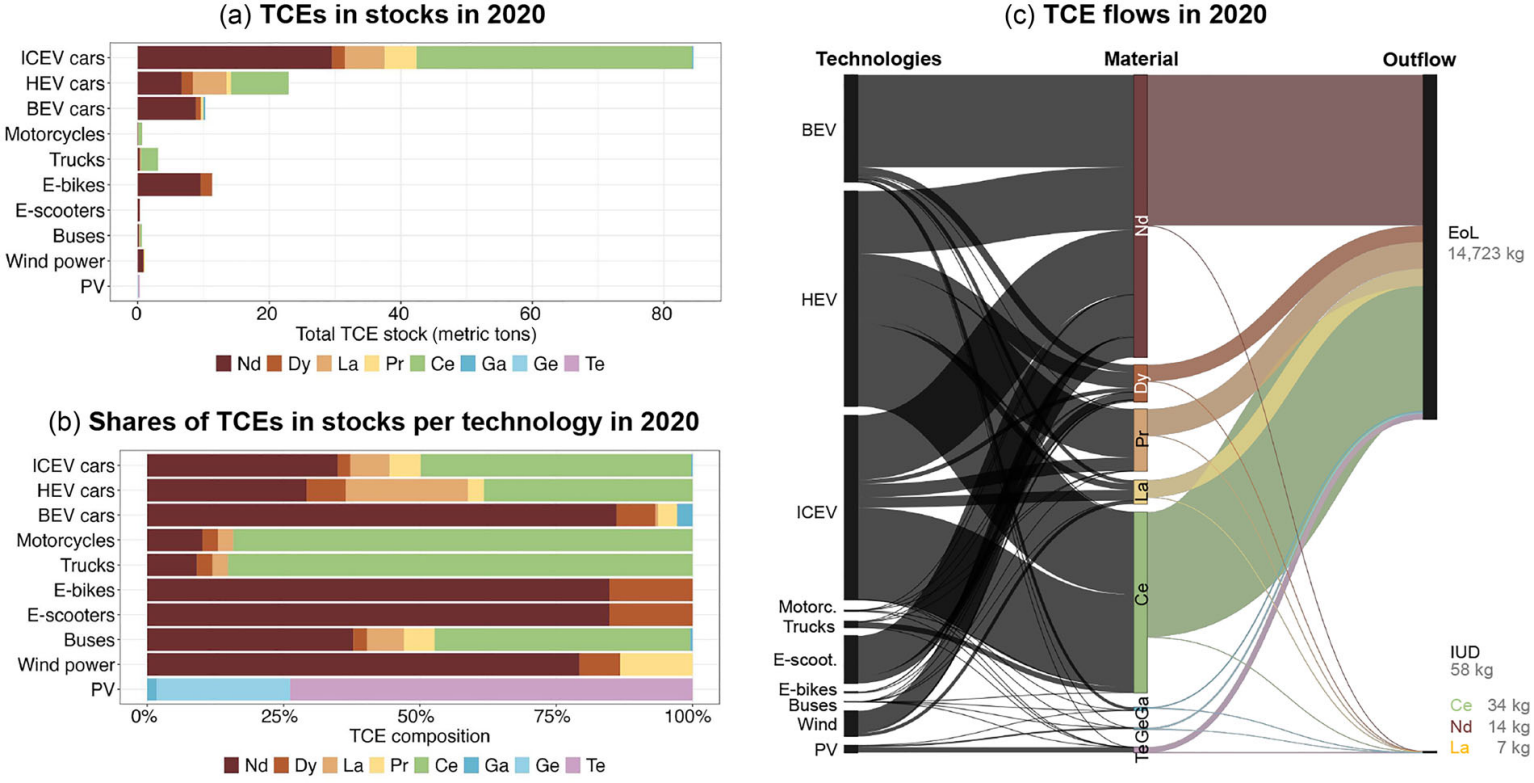
Mean‐estimate total technology‐critical element (TCE) stocks per technology (a), TCE composition of technology stocks (b) and Sankey diagram of mean‐estimate inflows per end‐use and TCE, and outflows (end‐of‐life and in‐use dissipation) in Vienna in 2020 (c). Note that of total TCE outflows only a fraction is attributed to in‐use dissipation. Underlying data for material stocks and flows as well as all technologies in natural numbers (e.g., number of vehicles) can be found in supplementary information S2. ICEV, internal‐combustion engine vehicle; HEV, hybrid‐electric vehicle; BEV, battery‐electric vehicle; Wind, wind power engines (all types); PV, photovoltaic (all types); IUD, in‐use dissipation. Nd, neodymium; Dy, dysprosium; La, lanthanum; Pr, praseodymium; Ce, cerium; Ga, gallium; Ge, germanium; Te, tellurium.

Total in‐use TCE stocks in vehicles, PV, and wind power amount to 135 [79–300] t within the city of Vienna (Figure 2). In‐use stocks are dominated by ICEV cars with 84 [42–229] t, accounting for 62% of total TCE in‐use stocks (Figure 2a). The majority of these are Ce (Figure 2b), primarily used in autocatalytic converters making up 19% of total TCE stocks. With 23 [19–31] t, HEV cars account for 17% of total TCE stocks. BEV cars currently constitute a small share of 8% of total TCE stocks, dominated by Nd, which is comparable to the TCE stock of e‐bikes.

The large importance of ICEV and HEV cars is also reflected in TCE inflows in the year 2020 (Figure 2c). However, with BEV cars becoming highly relevant for newly registered vehicles, BEV cars overtake ICEV vehicles in TCE consumption. While ICEV and HEV collectively account for 17 [12–34] kt, or 67% of total TCE inflows, BEV vehicles constitute 5 [4–5] kt, or 18%. Total inflows in 2020 amount to 26 [18–44] t. TCE‐wise, inflows in 2020 are dominated by Nd with 12 [9‐27] t, followed by Ce with 8 [5–10] t. Together they account for 78% of total TCE inflows. Outflows are dominated by EoL outflows with 15 [9–31] t, while in‐use dissipation accounts for only 0.4% of outflows, or 58 [30–118] kg in 2020.

### 3.2 Future TCE stocks and cumulative demand

**FIGURE 3:**
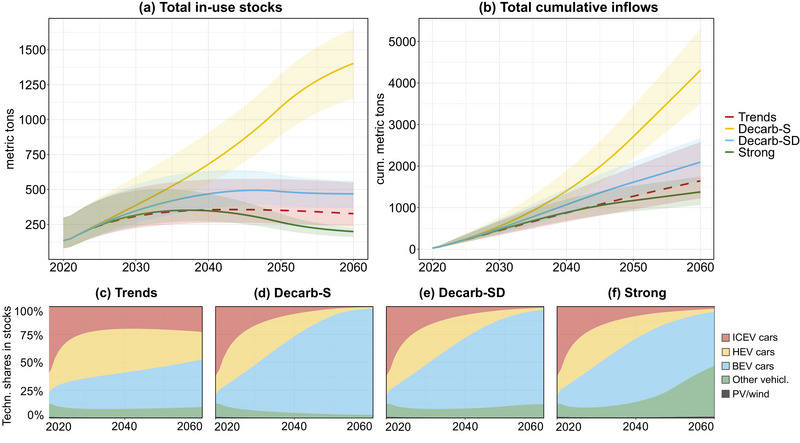
Total technology‐critical element (TCE) in‐use stocks (a), total cumulative TCE inflows (b), and technology shares in total TCE stocks in Vienna from 2020 to 2060 for the trends (c), Decarb‐S (d), Decarb‐SD (e), and “strong” scenario (f). Trends, scenario with continued trends; Decarb‐S, decarbonization with official targets with supply‐side measures; Decarb‐SD, decarbonization with official targets with combined supply‐ and demand‐side measures; Strong, scenario with measures going beyond official targets (see Section 2.3 for further information on scenarios). Underlying data can be found in supplementary information S2. ICEV, internal‐combustion engine vehicle; HEV, hybrid‐electric vehicle; BEV, battery‐electric vehicle; PV, photovoltaic.

Across the four scenarios, considerable differences are found regarding stock levels and cumulative inflows. In the trend‐continuation scenario, TCE stocks would increase to 355 [261–571] t in 2040 and 327 [235–551] t in 2060 (dashed line in Figure 3a). With decarbonization measures without demand reduction (Decarb‐S), material stocks would grow to 681 [541–907] t in 2040 and 1,402 [1,151–1,646] t in 2060, constituting a 10‐fold increase. This is predominantly driven by a gradual increase of a TCE‐intensive BEV fleet, which would account for 96% of total TCE stocks in 2060 (Figure 3d). A modal split shift introduced in the Decarb‐SD scenario would decrease in‐use stocks sharply to only 469 [370–563] t in 2060, a reduction of 73% compared to Decarb‐S in the same year. In a further‐reaching “strong” scenario, constituting a doubling of the car‐sharing rate and a 95% modal shift to public and active mobility, in‐use stocks are reduced to 199 [160–226] t in 2060, an 86% decrease compared to Decarb‐S. Total TCE stocks begin to stagnate around 2050 when new additions to the vehicle fleet are fully electrified. Reductions in car ownership and a shift toward public transportation modes are reflected in the reduced dominance of cars in total TCE stocks by 2060 (see Figure 3f).

Large quantities of material need to be mobilized to build up stocks and, once they have reached their EoL, to replace them. These can be expressed as cumulative inflows, that is, the sum of all annual inflows to societal stocks over multiple years (Figure 3b). Cumulative inflows do not strictly represent primary material demand as they may also include secondary material flows. In the “trends” scenario, cumulative inflows would reach 1.6 [1.2–2.6] kt within the 40‐year time frame from 2020 until 2060. With fleet electrification in the Decarb‐S scenario, this would more than triple to 4.3 [3.5–5.3] kt. With demand being reduced through a shift in the modal split in Decarb‐SD, cumulative inflows would decrease to 2.1 [1.6–2.7] kt by 2060, considerably closer to the levels in a scenario with continued trends and further decrease to 1.4 [1.1–1.8] kt in the “strong” scenario due to increased car sharing.

In Decarb‐S, with 3.5 [2.9–4.1] kt, new BEV cars consume almost 82% of all TCEs, the majority of which is Nd with 3.0 [2.6–3.4] kt (86% of all TCEs in BEV). In total, Nd makes up 3.4 [2.9–4.1] kt, or 79% of all cumulative inflows, followed by Dy with 0.3 [0.2–0.4] kt (7%). The dominance of these two elements can be ascribed to the widespread application of NdFeB magnets, especially in BEV electronic systems and motors, which consume 44% and 31% of total cumulative Decarb‐S scenario TCEs, respectively. Besides BEV cars, a significant share of TCEs is also being used in ICEV and HEV cars and e‐bikes, which collectively account for almost 7% of all TCE usage.

### 3.3 Future TCE in‐use dissipation and end‐of‐life flows

**FIGURE 4:**
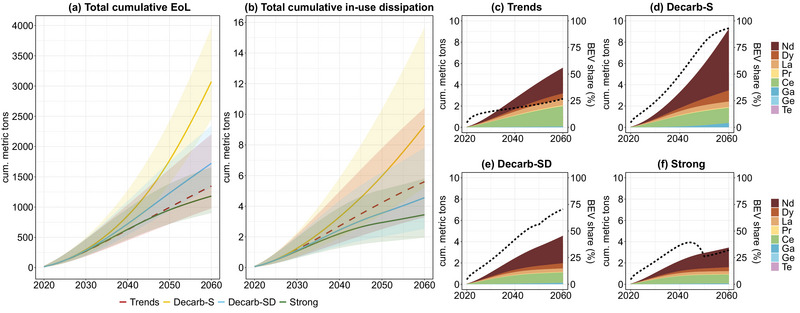
Total cumulative technology‐critical element (TCE) end‐of‐life (EoL) (a), in‐use dissipation (b), and detailed cumulative in‐use dissipation for all four scenarios (c–f) for neodymium (Nd), dysprosium (Dy), Lanthanum (La), praseodymium (Pr), cerium (Ce), gallium (Ga), germanium (Ge), and tellurium (Te) in Vienna (2020–2060). Share of battery‐electric vehicles (BEV) in total in‐use dissipation in percent as a dotted line. Trends, scenario with continued trends; Decarb‐S, decarbonization with official targets with supply‐side measures; Decarb‐SD, decarbonization with official targets with combined supply‐ and demand‐side measures; Strong, scenario with measures going beyond official targets (see Section 2.3 for further information on scenarios). Underlying data can be found in supplementary information S2.

In a trend‐continuing scenario, total cumulative EoL reaches 1.3 [1.0–2.2] kt by 2060 and would more than double to 3.1 [2.5–4.0] kt with only supply‐side measures in Decarb‐S (see Figure 4a). Combined supply‐ and demand‐side measures (Decarb‐SD) would reduce EoL to 1.7 [1.3–2.3] kt, while stronger combined measures would push cumulative EoL below “trends” levels, to 1.2 [0.9–1.7] kt. Most of EoL is dominated by Nd, which makes up 57% and 76% in the “trends” and Decarb‐S scenarios, respectively.

Cumulative in‐use dissipation in 2060 reaches 9.3 [5.2–15.7] t in the Decarb‐S scenario—almost twice the amount of the “trends” scenario with 5.6 [3.3–10.4] t (Figure 4b). Decarb‐SD scenario demand reductions decrease cumulative in‐use dissipation to 4.6 [2.5–7.8] t, to levels lower than in the “trends” scenario (Figure 4e). With far‐reaching measures (“strong” scenario), 3.4 [2.0–5.8] t, a third of Decarb‐S scenario in‐use dissipation, could be reached (Figure 4f). With 5.8 [3.3–10.0] t, or 62% of all dissipated TCEs in Decarb‐S, Nd dominates cumulative in‐use dissipation. This is the case for all prospective decarbonization scenarios (Figure 4d–f) predominantly driven by BEV cars (dotted line). In the trends scenario, with 1.9 [1.1–2.9] t, Ce cumulative mean‐estimate in‐use dissipation is only slightly lower than that of Nd with 2.4 [1.5–5.2] t. This can be attributed to Ce being essential for ICEVs, namely in autocatalytic converters in motor exhaust systems.

A summary table of cumulative EoL and in‐use dissipation of TCEs until 2060 can be found in Table 2. Nd dominates both cumulative EoL and in‐use dissipation in all scenarios. Ge and Te enter the EoL stage and are dissipated in comparably small amounts with less than 10 kg of in‐use dissipation from vehicles, PV, and wind power over the course of four decades. Cumulative EoL and in‐use dissipation for Nd, Dy, Ga, and Ge are higher in Decarb‐S compared to “trends” levels highlighting the demand‐increasing effect of fleet electrification. However, with modal split shifts in Decarb‐SD, EoL and in‐use dissipation of these TCEs are reduced. In the “strong” scenario, EoL of Dy and Pr and in‐use dissipation of Nd, Dy, and Ga are pushed below “trend” levels. All other TCEs are lower in the prospective scenarios compared to the “trends” scenario due to ICEV and HEV cars continuously being replaced by BEV cars.

**TABLE 2:** Mean‐estimate total cumulative end‐of‐life (EoL) and in‐use dissipation from 2020 to 2060 for all four scenarios in metric tons. Trends, scenario with continued trends; Decarb‐S, decarbonization with official targets with supply‐side measures; Decarb‐SD, decarbonization with official targets with combined supply‐ and demand‐side measures; Strong, scenario with measures going beyond official targets (see Section 2.3 for further information on scenarios). Refer to the supporting information for a disaggregation of material flows in yearly time‐steps.

	Cumulative EoL (t)				Cumulative in‐use dissipation (t)			
TCE	Trends	Decarb‐S	Decarb‐SD	Strong	Trends	Decarb‐S	Decarb‐SD	Strong
Nd	773	2,345	1,236	800	2.42	5.78	2.59	1.85
Dy	111	231	134	101	0.55	1.06	0.49	0.37
La	93	90	68	56	0.57	0.52	0.33	0.28
Pr	49	109	62	44	0.07	0.06	0.04	0.04
Ce	301	224	186	159	1.92	1.43	0.98	0.85
Ga	13	66	30	16	0.08	0.41	0.13	0.07
Ge	2	2	2	2	<0.01	<0.01	<0.01	<0.01
Te	5	5	5	5	<0.01	<0.01	<0.01	<0.01

*Note*: Trends, scenario with continued trends; Decarb‐S, decarbonization with official targets with supply‐side measures; Decarb‐SD, decarbonization with official targets with combined supply‐ and demand‐side measures; Strong, scenario with measures going beyond official targets (see Section 2.3 for further information on scenarios). Refer to the supporting information for a disaggregation of material flows in yearly time‐steps.

Abbreviations: EoL, end‐of‐life; TCE, technology‐critical element; Nd, neodymium; Dy, dysprosium; La, lanthanum; Pr, praseodymium; Ce, cerium; Ga, gallium; Ge, germanium; Te, tellurium.

Due to the modeling not being spatially explicit, no precise statements can be made regarding the spatial distribution of in‐use dissipation within the city. However, with location and movement disregarded, per square meter of city area, 13.5 [7.9–25.1] mg/m^2^ and 22.3 [12.6–37.7] mg/m^2^ of TCEs accumulate between 2020 and 2060 in the “trends” and Decarb‐S scenario, respectively. Even though TCEs are transported and deposited in the urban environment through abiotic elements, it is likely that concentrations are higher in the vicinity of those locations where TCEs are dissipated, for example, along high‐traffic roads (Mleczek et al., 2021). Relative to the urban population in 2060, cumulative in‐use dissipation reaches 2,382 [1,395–4,425] mg/cap in the “trends” scenario and 3,939 [2,223–6,660] mg/cap in the Decarb‐S scenario.

## 4 DISCUSSION

### 4.1 Climate change mitigation as a driver for sustainable resource use

The majority of investigated TCE demand, EoL, and in‐use dissipation can be expected due to the electrification of the Viennese vehicle fleet. PV panels and wind power play a secondary role, though the relevance of the energy technologies might increase if taking into consideration waste management sites. Even though conventional vehicles do contain TCEs, an adaptation of an electric vehicle fleet, as envisioned in the official city policies, would effectively double future TCE demand and urban accumulation in Vienna. A modal split shift from private passenger vehicles toward 85% public and active mobility decreases the need for vehicles and thus has the potential to reduce cumulative TCE inflows and in‐use dissipation considerably by 51% (Figure 4b), which might be achievable in an urban area such as Vienna with well‐developed public transport systems and high population density.

Due to the global transition of the transport and energy sectors toward novel technologies, sharp increases in demand as depicted in the Decarb‐S scenario could create significant supply chain bottlenecks (Ballinger et al., 2020; Schmid, 2020). Supply challenges include current levels of raw material production having to increase drastically to meet rising demand, as well as trade regulations such as export restrictions as have occurred in 2010 (Sprecher et al., 2015) and, more recently, in 2023. Even though thin‐film PV and wind power are not highly relevant in this case study area, this may not be the case at a European to global level where a widespread transition toward renewable energy will pose a challenge to sustainable resource supply, especially due to unpredictable geopolitical situations (Massari & Ruberti, 2013). With technologies such as PV constantly changing in material characteristics, modeling future technology changes is not possible and consequently not part of this study's scope. The results show what effect a shift from one to another existing technology can have on TCE demand. This highlights the need to take into consideration the criticality of materials used in future product design, both in terms of supply criticality, design for circularity (Graedel & Miatto, 2022), and toxicology. Hence, future design‐induced material composition changes in products can potentially reduce or increase the use and dissipation of TCEs.

### 4.2 TCE accumulation in the urban environment

Modeled EoL flows of TCEs are considerably larger than in‐use dissipation (Figure 2c and Table 2), indicating that the EoL phase, including collection, sorting, recycling, and final waste treatment are potentially highly relevant sources of TCE dissipation to the environment. Only some of these activities occur within the city of Vienna, with various collection and sorting facilities within city limits. Workers at these sites are therefore potentially exposed to significantly higher environmental TCE levels, especially if mechanical separation occurs (e.g., shredding and cutting). At an industrial scale, however, landfilling, scrapping, or recycling of EoL waste from vehicles, PV, and wind turbines occurs outside the city of Vienna. Consequently, most waste flows are managed beyond city limits or exported overseas for further use, as is common in the case of cars with three‐quarters of deregistered cars in Austria having been exported, mostly to Eastern Europe or Africa between 2006 and 2008 (Perpmer, 2016).

TCEs dissipating at sites in the vicinity of Vienna may find their way back to urban areas via waterways or other natural processes. Considering the highly increased risk of metals dissipating during waste management, it should be noted that in other cities, where waste management may exist within urban areas, EoL waste could contribute significantly to urban environmental TCE contamination. This is due to physically damaged EoL waste being commonly stored outdoors for longer periods of time exposing it to weather conditions and waste being subject to physical handling. Rainwater can thereby wash out TCEs from physically damaged technologies into the environment (Zapf‐Gottwick et al., 2015). Material loss rates for TCEs during storage at waste management facilities are reported to be significant (Wang et al., 2018). Currently, however, the recovery of these materials from tailings and the environment is highly challenging, especially due to their low concentrations in discarded products. Consequently, TCE recycling is associated with low yield and high costs (Balaram, 2019; Nuss & Blengini, 2018). To both close circularity gaps and reduce environmental accumulation, novel recycling technologies and improved design are key. To increase TCE circularity, recent advances in automated disassembly and chemistry‐based separation of TCEs from e‐waste (Balaram, 2019, 2024; Danouche et al., 2024) seem promising but are yet to be applied at large scale.

A small but not insignificant share of TCEs used in vehicles and renewable energy technologies is dissipated during the use phase. The vast majority of dissipated TCEs are emitted by vehicles due to corrosion, abrasion, or other causes, which means that the highest contamination through in‐use dissipation can be expected to be found along high‐traffic roads (Mirzaei Aminiyan et al., 2018; Pagano et al., 2019; Shajib et al., 2020). Previous studies showed that higher concentrations of heavy metals such as PGEs can be observed in commercial areas where vehicles are subject to mechanical activities such as repair (Trujillo‐González et al., 2016). However, with road dust and rainfall, TCE emissions may travel within and far outside of the city, for example, via waterways (Shajib et al., 2020; Yuan et al., 2018). Mobility infrastructure currently makes up 15% of the total surface area of Vienna (Magistrat der Stadt Wien, 2023). With TCEs emitted on sealed surfaces being further dispersed by rainfall (Lerat‐Hardy et al., 2022), TCE pollutants can reach soils, parks, and urban gardening lots, as well as waterways. A potential pathway of TCE exposure is also through food consumption, for example, through the consumption of locally grown vegetables (Arciszewska et al., 2022). Urban allotment gardening is widespread in Vienna, in some cases in close proximity to high‐traffic areas, both in the urban center of Vienna as well as its periphery. Although relatively low levels of REEs in consumed vegetables do not necessarily present a health hazard, continuous long‐term exposure was shown to be a greater potential danger to health, especially in children (Arciszewska et al., 2022; Zhuang et al., 2017).

Even though in‐use dissipation represents only a small share of total outflows, over time, such dissipation possibly accumulates to relevant scales. The precise extent as to how much TCEs are dissipated to the environment in total (i.e., beyond the technologies covered herein) and to what extent urban environmental accumulations push concentrations above certain thresholds is not yet clearly understood but requires further scientific attention (Balaram, 2019). First efforts have been performed to measure TCE accumulation from plant leaves and water samples taken from the city of Vienna (Trimmel et al., 2024). Furthermore, the fact that losses to the environment are practically unrecyclable (Zimmermann & Gößling‐Reisemann, 2013) highlights the need to reflect upon and reduce the chance of in‐use dissipation in the product design stage.

### 4.3 Toxicological effects of TCE exposure

We find that decarbonizing only through supply‐side technology measures (e.g., BEV cars and PV panels) could lead to a sharp increase in dissipation to the urban environment (Table 2). Without demand‐reducing measures, Nd, the most common TCE, could increase by 139% by 2060, compared to the “trends” scenario. Once TCEs are dissipated to the environment, they are mobilized into the air and waterways, accumulate in the ground and plants, and can reach the human body via dust, water, or locally grown food from urban gardening.

For evaluating the toxicity of TCEs, their particular chemical species in environmental matrices and their bioavailability need to be taken into account (Qvarforth et al., 2022). Among other possible health hazards, exposure to Nd compounds has been associated with increased risks of lung embolism, liver damage, and low cell proliferation (Brouziotis et al., 2022; Filella & Rodríguez‐Murillo, 2017; Hua et al., 2019; Shin et al., 2019), while data suggest that Dy exposure is related to damage to mitochondrial DNA in umbilical cord blood, potentially harming newborns (Liu et al., 2021). A detailed overview of existing toxicological data for the main TCEs is provided in the supporting information.

Although single elements have been shown to affect human health directly and indirectly, TCE levels in the environment are not of high public concern now. Health and safety regulations, particularly in the context of occupational and environmental exposure, play a crucial role in mitigating risks and safeguarding public health. While some countries, such as Austria, have well‐established regulatory frameworks in this regard, global disparities exist, highlighting the need for harmonized standards and enhanced regulatory enforcement. Notably, significant future accumulation can be expected with increasing use of TCEs, posing a future public health hazard of a so far unknown scale, especially as transgenerational transmission of genotoxic effects due to environmental exposure may occur. Nevertheless, dissipation of other materials—often occurring in higher concentrations—from conventional technologies may likewise be associated with possible health hazards and therefore warrant further monitoring.

Understanding the mechanisms underlying such effects is essential for evaluating long‐term health implications and implementing preventive measures to mitigate adverse outcomes across generations. The current lack of a clear understanding of movement and the health risks associated with TCEs warrants further investigation. A comprehensive understanding of the chemical properties and toxicity of environmental contaminants is essential for evaluating their impacts on human health and the environment.

### 4.4 Limitations

The model developed in this study quantifies current and future TCE stocks and flows, including in‐use dissipation at the urban level. However, data limitations in five areas warrant future research. First, a lifetime‐based leaching model as applied herein is a simplified representation of reality but necessary in the face of a lack of age‐cohort‐specific vehicle data at the city level.

Second, due to the low number of available in‐use dissipation factors, the same rates were used for various TCEs within the same technology. Consequently, this study provides a first estimation of in‐use dissipation at an urban level. With more empirical data on in‐use dissipation, the degree to which stock‐driven models can realistically predict material in‐use dissipation would increase significantly.

Third, future technological changes are difficult to predict. Therefore, a limited timeframe of 40 years was used, roughly corresponding to two to three product lifetimes, disregarding technological changes beyond a gradual fleet replacement. Therefore, MI factors and in‐use dissipation rates were kept constant.

Fourth, other sources of TCE in‐use dissipation where dissipation causality is currently not clearly understood were not modeled herein. This includes various electronic appliances.

Last, the actual degree to which the urban population is exposed to TCEs cannot yet effectively be quantified. Beyond the in‐use dissipation quantified herein, TCE pollution is likely to be increased by flows from outside the city boundaries. At the same time, some TCEs dissipated within the city will be transported beyond city limits. This movement of dissipated material complicates the use of cumulative flows, as local cumulative in‐use dissipation may not strictly correspond to local concentration increases. However, with annual in‐use dissipation not being as relevant in scale and for a lack of data regarding environmental transportation of dissipated TCEs, this study nevertheless focuses on cumulative in‐use dissipation to approximate the overall TCE in‐use dissipation capacity of the city regardless of the exact final location of dissipated material. While some qualitative information on possible pathways of TCE consumption exists, these are likewise not quantifiable with the data at hand, arguing for the need for an extension of both geographical system boundaries and the life cycle analyzed, including waste treatment. The authors stress the need for further interdisciplinary research in this regard.

## 5 CONCLUSIONS

Fleet electrification, a modal split shift, and a transition toward renewable energy are core aspects of the Smart City Strategy Vienna. Though vital for reducing greenhouse gas emissions and staying within carbon budgets, our results show that such measures would significantly increase TCE stocks and consequently in‐use dissipation, unless demand‐side measures resulting in strong modal shifts and traffic volume reduction are implemented as well. Even then, substantial end‐of‐life amounts of TCEs can be expected, from which significant dissipation to the environment is likely to occur, depending on the type of end‐of‐life treatment.

Current scientific knowledge on the way that TCEs are used, dissipated, and transported in the environment and how they might reach the human body is limited. However, fleet electrification and expanding renewable energy supply are integral aspects of all sustainable transition roadmaps, such as the EU 2050 long‐term strategy. Consequently, environmental levels can be expected to increase. Accurate data acquisition and measurement methodologies are fundamental for assessing human exposure to environmental contaminants.

For the sake of putting TCE in‐use dissipation from emerging technologies into perspective, it should be noted that conventional technologies can be expected to contribute to urban environmental accumulation of materials to a significant degree as well. Materials present in comparably larger quantities in conventional technologies may, too, be associated with environmental and health risks. Nevertheless, currently, TCEs remain particularly understudied and were consequently the focus of this study.

Future interdisciplinary research should focus on limiting the dissipation of TCE in use and exploring the pathways through which it might reach humans via different ecological compartments. From a sustainability perspective, this approach will help identify strategies to mitigate public health risks associated with TCE exposure, both in the near term and for future generations.

## AUTHOR CONTRIBUTIONS

**André Baumgart**: Conceptualization; methodology; software; visualization; formal analysis; investigation; resources; writing—original draft. **Daniela Haluza**: Investigation; resources; writing—original draft; review and editing; funding acquisition. **Thomas Prohaska**: Review and editing; funding acquisition. **Simone Trimmel**: Review and editing. **Ulrike Pitha**: Review and editing; funding acquisition. **Johanna Irrgeher**: Conceptualization; resources; review and editing; supervision; project administration; funding acquisition. **Dominik Wiedenhofer**: Conceptualization; methodology; investigation; resources; writing—review and editing; supervision; project administration; funding acquisition.

## CONFLICT OF INTEREST STATEMENT

The authors declare no conflict of interest.

## Supporting information




**Supporting Information S1** This supporting information provides values and data sources for additional model inputs including in‐use dissipation rates, lifetime assumptions, and various inventory data. Furthermore, this supporting information explains in more detail scenario differences and additional health implications.

**Supporting Information S2** This supporting information provides material intensity factors, inventory data, detailed results for TCE stocks and flows per technology and scenario, and underlying data of manuscript figures.

## Data Availability

The data that supports the findings of this study are available in the supporting information of this article.
