# Supplementary material for: In-use dissipation of technology-critical elements from vehicles and renewable energy technologies in Vienna, Austria: A public health matter?
Source: J Ind Ecol. 2024 Oct 16;28(6):1857–70. doi: 10.1111/jiec.13571 (PMC11667654; doi:10.1111/jiec.13571)
Supplement: Supplementary file 1 — Supporting Information S1 This supporting information provides values and data sources for additional model inputs including in-use dissipation rates, lifetime assumptions, and various inventory data. Furthermore, this supporting information explains in more detail scenario differences and additional health implications. [file 44498_2024_2806038_MOESM1_ESM.pdf]

SUPPORTING INFORMATION FOR:

Baumgart, A., Haluza, D., Prohaska, T., Trimmel, S., Pitha, U., Irrgeher, J. & Wiedenhofer, D. (2024.) In-use dissipation of technology-critical elements from vehicles and renewable energy technologies in Vienna, Austria: A public health matter? *Journal of Industrial Ecology.*

Summary

This supporting information provides values and data sources for additional model inputs including in-use dissipation rates, lifetime assumptions, and various inventory data. Furthermore, this supporting information explains in more detail scenario differences.

1. Scenarios

Trends scenario

In the trend-continuing scenario, henceforth referred to as ‘trends’, historic vehicle fleet and PV installed capacity additions were coupled with historic pkm and tkm from statistics with trends then forecasted. Pkm in turn were forecasted to 2060 via coupling with Statistik Austria population forecasts (Statistik Austria, 2023). Technology shares were kept static based on shares of the last available year, e.g., the share of electric vehicles in the total vehicle fleet does not change in the ‘trends’ scenario.
The installed capacity of wind power is expanded until 2030 followed by a gradual decline to only replacement in 2040. This assumption is based on the limited space for wind power expansion within Vienna city limits.

Three additional prospective scenarios were developed and compared against the ‘trends’ scenario: Decarb-S, Decarb-SD, and a ‘strong’ scenario. Target values such as concrete future fleet composition and installed capacity, which were derived from literature, were used and linearly interpolated to.

Decarb-S scenario (decarbonisation with supply-side measures)

A Decarb-S scenario reflects a decarbonized future based on targets currently envisioned in the Smart City strategy (Stadt Wien, 2019). This strategy includes an electrification of all newly registered personal vehicles in Vienna by 2050, and an increase of the installed PV capacity in Vienna to reach 800 MW by 2040.

Decarb-SD scenario (decarbonisation with combined supply- & demand-side measures)

To make high-impact policy measures more discernable, a Decarb-S scenario (decarbonization with combined supply- and demand-side measures) was added. Beyond an electrification of newly registered personal vehicles, Decarb-S includes the official goal

of achieving a 85% share of public and active mobility in the modal split by 2050 (Stadt Wien, 2019), thereby integrating supply- and demand-side measures.

Strong scenario (decarbonisation with further-reaching measures)

To explore an even more ambitious scenario, referred to as 'strong', we go beyond targets defined in the Smart City strategy and include further-reaching measures based on own assumptions. Besides an increase of the share of public and active mobility in the total modal split to 95% by 2050, car sharing is assumed to double in the same timeframe. Furthermore, not only personal vehicles, but the entire vehicle fleet is assumed to be electrified by 2060. Installed capacity of PV is assumed to double by 2060 compared to the installed capacity of Decarb-S.

Wind power expansion

For wind power expansion no clear official targets could be identified. However, to distinguish between the scenarios, different pathways of installed capacity expansion were assumed. Due to the limited area in Vienna, limits to expansion were set but gradually pushed forward with increasing scenario intensity. Consequently, wind power expansion was assumed until 2040, 2050 and 2060, for Decarb-S, Decarb-SD and the strong scenario respectively.

Technology shares

Note that for both PV and wind power, baseline year technology shares in newly installed capacity (CIGS, CdTe and a-Si for PV and geared vs. direct drive for wind power) were kept constant throughout the scenarios. For vehicles, technology shares are driven by either constant technology shares (Trends scenario) or by a decarbonisation (phase-out internal-combustion engine vehicles (ICEV) and replacement with electric vehicles. Electric vehicles are either battery-electric (BEV) or hybrid-electric (HEV) vehicles with HEVs gradually replacing BEV vehicles. All electric vehicles are assumed to be BEVs by 2050.

2. Additional model details

Material stocks are calculated by multiplying the inventory, or function unit (number of vehicles, installed capacity), in a given year with the respective material intensity (MI) factor. To calculate the inventory in a given year, the annual inflow is added to and the annual EoL outflow is deducted from the preceding year's inventory. Inflows are calculated as the difference between two consecutive years' inventories plus the EoL flow that needs to be replaced. EoL outflows are in turn calculated by dividing the annual stock with the respective lifetime, based on a leaching lifetime model approach (van der Voet et al., 2002). The following two equations summarize the relationship between stocks and flows.

Equation SI-1 was used to calculate the TCE *inflow*, or additions to stock. *inflow* is the annual change of TCE stocks (*MS)* plus the replacement of stocks having reached their end-of-life (*EOL*). *e* is the investigated TCE, *c* is the technology or technology component, and *t* is the corresponding year*, and t-1* refers to the stocks or flows or the preceding year.

$$inflow_{e,t} = \sum_{c} \left[MS_{e,c,t} - MS_{e,c,t-1} + EOL_{e,c,t-1} \right] \quad \text{(Equ. SI-1)}$$

Similarly, end-of-life outflows (*EOL*) were calculated as described in Equation SI-2. *EOL* is calculated by dividing the material stock (*MS*) in a given year by the technology-specific lifetime (*LT*).

$$EOL_{e,t} = \sum_{c} \left[\frac{MS_{e,c,t}}{LT_c} \right] \quad \text{(Equ. SI-2)}$$

Table SI-1 provides an overview of the units of the variables used in the manuscript equations for the calculation of TCE stocks and flows.

Table SI-1 Model variables. Note that abbreviations correspond to variables used in Equations 1 and 2 in the manuscript and variables used in Equations SI-1 and SI-2 as defined above.

Abbreviation	Variable	Unit
MS	Material stock	kg
IUD	In-use dissipation to the environment	kg/year
inflow	Inflows (additions to and replacements of stocks)	kg/year
EOL	End-of-life outflows from stocks	kg/year
INV	Technology inventory (fleet size, installed capacity)	number of vehicles or MW
IUDR	Technology- and TCE-specific in-use dissipation rate	kg/km or kg/year of LT
LT	Technology-specific lifetime	years
LTvkm	Average vehicle-km per lifetime per technology	vkm/LT
vkm	Vehicle-km travelled per year	vkm/year
IC	Installed capacity per technology	MW

3. Data sources

Table SI-2 gives an overview of data sources various data inputs used in the modelling of TCE stocks and flows in Vienna, including in-use dissipation rates, lifetimes, occupancy rates, energy technology market shares, installed capacity, vehicle fleet size, traffic volume, and population data.

Table SI-2 Overview of model inputs and data sources per technology and component (where applicable), as well as sources for inventories and additional data.

Data type	Technology	Component	Value	Unit	Sources
In-use dissipation (IUD)	Cars	Autocatalytic converter	2%	% lost/LT	Ciacci et al., 2015
		Motor	2%	% lost/LT	
		NiMH battery	2%	% lost/LT	
		Brake pads/tires	5%	% lost/LT	Mleczek et al., 2021
	Other vehicles, PV, wind power	Total	2%	% lost/LT	*Own assumption based on cars*
Lifetime (LT)	Cars	Total	10 (200.000)	years (km)/LT	Mleczek et al., 2021
		Brake pads	2 (40.000)		
		Tires/treads	4 (80.000)		
	Motorcycles	Total	10 (80.000)		Carranza et al., 2022
	Trucks	Total	15 (400.000)		Iyer et al., 2023
	E-bikes/e-scooters	Total	10 (15.000)		Brightside, 2022; Rizos et al., 2022
	Buses	Total	12 (480.000)		Luo et al., 2019
Share of installed PV panels assumed to break due to any causes (weather, faulty assembly)	PV		1%	% of installed capacity	Sinha et al., 2012
Occupancy rates	Cars		1.4	passenger/ vehicle/ trip	Maier, 2015
	Buses		50		Knoflacher & Pfaffenbichler, 2002
	Two-wheeled vehicles		1.0		*Own assumption*
Share of HEV cars using NiMH batteries [1]	HEV cars		50%	% of fleet	Barteková, 2016
Market share [2]	PV				Cichy, 2017; Biermayr et al., 2021; Fraunhofer Institute for Solar Energy Systems, 2023
	Wind power				AEA, 2021
Installed capacity [2]	PV				PV Austria, 2024
	Wind power				IG Windkraft, 2023
Vehicle fleet [2]					Gassner et al., 2020
Traffic volume [2]					
Population [2,3]					Statistik Austria, 2023

[1] It is assumed that NiMH are continuously replaced by li-ion batteries with complete substitution by 2050.
[2] Historic data
[3] Projected data

Please refer to the supporting information S2 for sources of material intensity (MI) factors. In addition to 75 individual MI factors used for TCE contents in various technologies and sub-components, 13 sources were used to disaggregate neodymium-iron-boron (NdFeB) permanent magnet mass into individual TCEs.

4. Additional health implications

In the following, an overview of existing toxicological data on the investigated elements is given. Due to the low quantity of modeled Ge and Te in-use dissipation (manuscript Table 2), the following overview of toxicological effects focuses on the remaining six modeled TCEs: Nd, Dy, La, Pr, Ce and Ga.

Neodymium

Nd compounds, which, driven by use in NdFeB magnets, account for most of the in-use dissipation in this study, have been associated with risks of lung embolism, liver damage, fever, chills, myalgia and spastic abdominal pain (Filella & Rodríguez-Murillo, 2017; Shin et al., 2019). Furthermore, Nd was shown to be cytotoxic causing low cell proliferation (Hua et al., 2019). Particles from NdFeB permanent magnets are cytotoxic against human oral mucosal fibroblasts and were shown to be capable of amplifying oxidative stress in lung cancer cells (Brouziotis et al., 2022).

Dysprosium

Exposure of women to Dy during pregnancy was found to be related to damage to mitochondrial DNA in umbilical cord blood, thus suggesting sensitivity to Dy in newborns (Liu et al., 2021) In-vitro tests showed that Dy ions block ATPase in human cells, disrupting biochemical processes in the human body (Rim, 2016). Like Nd, the majority of Dy dissipation comes from NdFeB magnets in BEV cars and other technologies.

Lanthanum

Prolonged La exposure can increase the risk of pneumoconiosis as well as acting cytotoxic, including inducing apoptosis in human cell lines (Rim, 2016; Wu et al., 2020). In addition, La can potentially enhance reactive oxygen species (ROS) production in lymphatic cells (Brouziotis et al., 2022).

Praseodymium

Pr has low solubility and a low clearing rate that could augment its toxic effects (Feyerabend et al., 2010). A minor cytotoxic effect in human cells could be demonstrated in vitro with human osteosarcoma cells showing reduced cell viability after treatment with Pr. For instance, Pr is hepatotoxic by inducing fatty liver (Nakamura et al., 1997).

Cerium

Exposure to Ce compounds, used mainly within autocatalytic converters, can cause health concerns, e.g., increased levels of proinflammatory cytokines in the brain and liver (Pagano et al., 2015). Skin irritation and lesions, pneumoconiosis, acute myocardial infarction in

elderly people, as well as interference with the cell machinery were found to be associated with Ce exposure (Steiner et al., 2012; Gwenzi et al., 2018; Shin et al., 2019). Furthermore, Ce, as well as La can increase the risk of infertility and certain cancers (Brouziotis et al., 2022).

Gallium

Cellular iron uptake can be inhibited by stable complexes of transferrin and Ga, thereby disturbing cellular proliferation. Furthermore, Ga can inhibit protein synthesis and cause gene expression alterations and induce heme oxygenase stress proteins, while also causing testicular toxicity and negatively impacting the immune system (White & Shine, 2016).

5. References

AEA. (2021). *Klima und Energie: Wissen kompakt*. Austrian Energy Agency.

Barteková, E. (2016). The Role of Rare Earth Supply Risk in Low-Carbon Technology Innovation. In *Rare Earths Industry* (pp. 153–169). Elsevier. https://doi.org/10.1016/B978-0-12-802328-0.00010-3

Biermayr, P., Dißauer, C., Eberl, M., Enigl, M., Fechner, H., Fürnsinn, B., Jaksch-Fliegenschnee, M., Leonhartsberger, K., Moidl, S., Prem, E., Schmidl, C., Strasser, C., Weiss, W., Wittmann, M., Wonisch, P., & Wopienka, E. (2021). *Innovative Energietechnologien in Österreich: Marktentwicklung 2020* (p. 273). https://nachhaltigwirtschaften.at/resources/iea_pdf/marktentwicklung-2020_web.pdf

Brightside. (2022). *How long do e-scooters last? A new approach to measuring micromobility lifespan*. https://www.brightside.city/work/lifespan-report

Brouziotis, A. A., Giarra, A., Libralato, G., Pagano, G., Guida, M., & Trifuoggi, M. (2022). Toxicity of rare earth elements: An overview on human health impact. *Frontiers in Environmental Science*, *10*, 948041. https://doi.org/10.3389/fenvs.2022.948041

Carranza, G., Do Nascimiento, M., Fanals, J., Febrer, J., & Valderrama, C. (2022). Life cycle assessment and economic analysis of the electric motorcycle in the city of Barcelona and the impact on air pollution. *Science of The Total Environment*, *821*, 153419. https://doi.org/10.1016/j.scitotenv.2022.153419

Ciacci, L., Reck, B. K., Nassar, N. T., & Graedel, T. E. (2015). Lost by Design. *Environmental Science & Technology*, *49*(16), 9443–9451. https://doi.org/10.1021/es505515z

Cichy, M. (2017). *Kritische Mineralische Ressourcen von Photovoltaik-Dünnschicht-Technologien. Eine Analyse unter Betrachtung der technologischen und marktwirtschaftlichen Entwicklung*. BOKU.

Feyerabend, F., Fischer, J., Holtz, J., Witte, F., Willumeit, R., Drücker, H., Vogt, C., & Hort, N. (2010). Evaluation of short-term effects of rare earth and other elements used in magnesium alloys on primary cells and cell lines☆. *Acta Biomaterialia*, *6*(5), 1834–1842. https://doi.org/10.1016/j.actbio.2009.09.024

Filella, M., & Rodríguez-Murillo, J. C. (2017). Less-studied TCE: Are their environmental concentrations increasing due to their use in new technologies? *Chemosphere*, *182*, 605–616. https://doi.org/10.1016/j.chemosphere.2017.05.024

Fraunhofer Institute for Solar Energy Systems. (2023, February 21). *Photovoltaics Report*. https://www.ise.fraunhofer.de/content/dam/ise/de/documents/publications/studies/Photovoltaics-Report.pdf

Gassner, A., Lederer, J., & Fellner, J. (2020). Material stock development of the transport sector in the city of Vienna. *Journal of Industrial Ecology*, *24*(6), 1364–1378. https://doi.org/10.1111/jiec.13024

Gwenzi, W., Mangori, L., Danha, C., Chaukura, N., Dunjana, N., & Sanganyado, E. (2018). Sources, behaviour, and environmental and human health risks of high-

technology rare earth elements as emerging contaminants. *Science of the Total Environment*, *636*, 299–313. https://doi.org/10.1016/j.scitotenv.2018.04.235

Hua, Q., Chen, Y., Liu, Y., Li, M., Diao, Q., Xue, H., Zeng, H., Huang, L., & Jiang, Y. (2019). Circular RNA 0039411 Is Involved in Neodymium Oxide-induced Inflammation and Antiproliferation in a Human Bronchial Epithelial Cell Line via Sponging miR-93-5p. *Toxicological Sciences*, *170*(1), 69–81. https://doi.org/10.1093/toxsci/kfz074

IG Windkraft. (2023, December 1). *Beschleunigung der Windgeschwindigkeit 2023?* https://www.igwindkraft.at/mmedia/download/2023.01.11/1673456390551631.pdf

Iyer, R. K., Kelly, J. C., & Elgowainy, A. (2023). Vehicle-cycle and life-cycle analysis of medium-duty and heavy-duty trucks in the United States. *Science of The Total Environment*, *891*, 164093. https://doi.org/10.1016/j.scitotenv.2023.164093

Knoflacher, H., & Pfaffenbichler, P. (2002). Busbuchten – eine sachlich nicht zu rechtfertigende Benachteiligung des öffentlichen Verkehrs. *Union Internationale Des Transports Publics*, *2*, 42–45.

Liu, Y., Wu, M., Song, L., Bi, J., Wang, L., Chen, K., Liu, Q., Xiong, C., Cao, Z., Li, Y., Xia, W., Xu, S., & Wang, Y. (2021). Association between prenatal rare earth elements exposure and premature rupture of membranes: Results from a birth cohort study. *Environmental Research*, *193*, 110534. https://doi.org/10.1016/j.envres.2020.110534

Luo, H., Kou, Z., Zhao, F., & Cai, H. (2019). Comparative life cycle assessment of station-based and dock-less bike sharing systems. *Resources, Conservation and Recycling*, *146*, 180–189. https://doi.org/10.1016/j.resconrec.2019.03.003

Maier, P. (2015). *Wachsende Fahrradnutzung in Wien und ihre Relevanz für Klima und Gesundheit* (165; Social Ecology Working Paper). Institute of Social Ecology. https://boku.ac.at/fileadmin/data/H03000/H73000/H73700/Publikationen/Working_Papers/working-paper-165-web.pdf

Mleczek, P., Borowiak, K., Budka, A., Szostek, M., & Niedzielski, P. (2021). Possible sources of rare earth elements near different classes of road in Poland and their phytoextraction to herbaceous plant species. *Environmental Research*, *193*, 110580. https://doi.org/10.1016/j.envres.2020.110580

Nakamura, Y., Tsumura, Y., Tonogai, Y., Shibata, T., & Ito, Y. (1997). Differences in Behavior among the Chlorides of Seven Rare Earth Elements Administered Intravenously to Rats. *Fundamental and Applied Toxicology*, *37*, 106–116.

Pagano, G., Guida, M., Tommasi, F., & Oral, R. (2015). Health effects and toxicity mechanisms of rare earth elements-Knowledge gaps and research prospects. *Ecotoxicology and Environmental Safety*, *115*, 40–48. https://doi.org/10.1016/j.ecoenv.2015.01.030

PV Austria. (2024). *Photovoltaik in Wien*. https://pvaustria.at/dashboard/wien/

Rim, K.-T. (2016). Effects of rare earth elements on the environment and human health: A literature review. *Toxicology and Environmental Health Sciences*, *8*(3), 189–200. https://doi.org/10.1007/s13530-016-0276-y

Rizos, V., Righetti, E., & Kassa, A. (2022). *Developing a supply chain for recycled rare earth permanent magnets in the EU: challenges and opportunities* (CEPS In-Depth Analysis). CEPS.

Shin, S.-H., Kim, H.-O., & Rim, K.-T. (2019). Worker Safety in the Rare Earth Elements Recycling Process From the Review of Toxicity and Issues. *Safety and Health at Work*, *10*(4), 409–419. https://doi.org/10.1016/j.shaw.2019.08.005

Sinha, P., Balas, R., Krueger, L., & Wade, A. (2012). Fate and transport evaluation of potential leaching risks from cadmium telluride photovoltaics. *Environmental Toxicology and Chemistry*, *31*(7), 1670–1675. https://doi.org/10.1002/etc.1865

Stadt Wien. (2019). *Smart City Wien Rahmenstrategie 2019-2050* (p. 172).

Statistik Austria. (2023). *Population projections for Austria and federal states* [dataset]. https://www.statistik.at/en/statistics/population-and-society/population/demographische-prognosen/population-projections-for-austria-and-federal-states

Steiner, S., Mueller, L., Popovicheva, O. B., Raemy, D. O., Czerwinski, J., Comte, P., Mayer, A., Gehr, P., Rothen-Rutishauser, B., & Clift, M. J. D. (2012). Cerium dioxide nanoparticles can interfere with the associated cellular mechanistic response to diesel exhaust exposure. *Toxicology Letters*, *214*(2), 218–225. https://doi.org/10.1016/j.toxlet.2012.08.026

van der Voet, E., Kleijn, R., Huele, R., Ishikawa, M., & Verkuijlen, E. (2002). Predicting future emissions based on characteristics of stocks. *Ecological Economics*, *41*(2), 223–234. https://doi.org/10.1016/S0921-8009(02)00028-9

White, S. J. O., & Shine, J. P. (2016). Exposure Potential and Health Impacts of Indium and Gallium, Metals Critical to Emerging Electronics and Energy Technologies. *Current Environmental Health Reports*, *3*(4), 459–467. https://doi.org/10.1007/s40572-016-0118-8

Wu, J., Yang, J., Yu, M., Sun, W., Han, Y., Lu, X., Jin, C., Wu, S., & Cai, Y. (2020). Lanthanum chloride causes blood-brain barrier disruption through intracellular calcium-mediated RhoA/Rho kinase signaling and myosin light chain kinase. *Metallomics*, *12*(12), 2075–2083. https://doi.org/10.1039/d0mt00187b
